# Research Progress on the Ability of Astragaloside IV to Protect the Brain Against Ischemia-Reperfusion Injury

**DOI:** 10.3389/fnins.2021.755902

**Published:** 2021-11-16

**Authors:** Xianhui Kang, Shuyue Su, Wandong Hong, Wujun Geng, Hongli Tang

**Affiliations:** ^1^Department of Anesthesiology, The First Affiliated Hospital of Wenzhou Medical University, Wenzhou, China; ^2^Department of Anesthesiology, The First Affiliated Hospital, Zhejiang University School of Medicine, Hangzhou, China; ^3^Wenzhou Medical University, Wenzhou, China; ^4^Department of Gastroenterology and Hepatology, The First Affiliated Hospital of Wenzhou Medical University, Wenzhou, China; ^5^Wenzhou Key Laboratory of Perioperative Medicine, Wenzhou, China

**Keywords:** Radix Astragali, astragaloside-IV, stroke, cerebral protection, ischemia reperfusion

## Abstract

Stroke, a disease with a sudden onset and high morbidity and mortality rates, is difficult to treat in the clinic. Traditional Chinese medicine has become increasingly widely used in clinical practice. Modern pharmacological studies have found that Radix Astragali has a variety of medicinal properties, i.e., immunoregulatory, antioxidative, anti-cancer, anti-diabetes, myocardial protective, hepatoprotective, and antiviral functions. This article reviews the protective effect and mechanism of astragaloside IV, which is extracted from Radix Astragali, on stroke, discusses the cerebroprotective effect of astragaloside IV against ischemia-reperfusion-related complications, offers insight into research prospects, and expands the idea of integrating traditional Chinese and Western medicine treatment strategies and drugs to provide a theoretical reference for the clinical treatment of cerebral ischemia-reperfusion injury and the improvement of stroke prognosis.

## Introduction

Stroke has a high incidence and is the main cause of death worldwide. Restoration of cerebral blood supply as soon as possible, i.e., reperfusion, is currently the best method for protecting the brain against ischemic injury. Recombinant tissue plasminogen activator (rtPA), which is the only FDA-approved treatment for ischemic stroke, has a significant time-dependent therapeutic effect. rtPA is most effective when administered within 90 min before symptoms appear (Lansberg et al., 2009), and the prognosis of elderly patients and patients with severe stroke treated with rtPA is still poor (Saposnik et al., 2013). Due to the injury caused by reperfusion and the narrow 4.5 h therapeutic window of rtPA, few patients are suitable for rtPA treatment. In clinical practice, only approximately 3% of patients can be treated with rtPA (Armstead et al., 2010). If rtPA is administered after beyond the therapeutic window thrombolysis, the risk of hemorrhagic transformation and fatal edema due to cerebral ischemia-reperfusion injury is elevated (Shafi and Levine, 2010).

**FIGURE 1:**
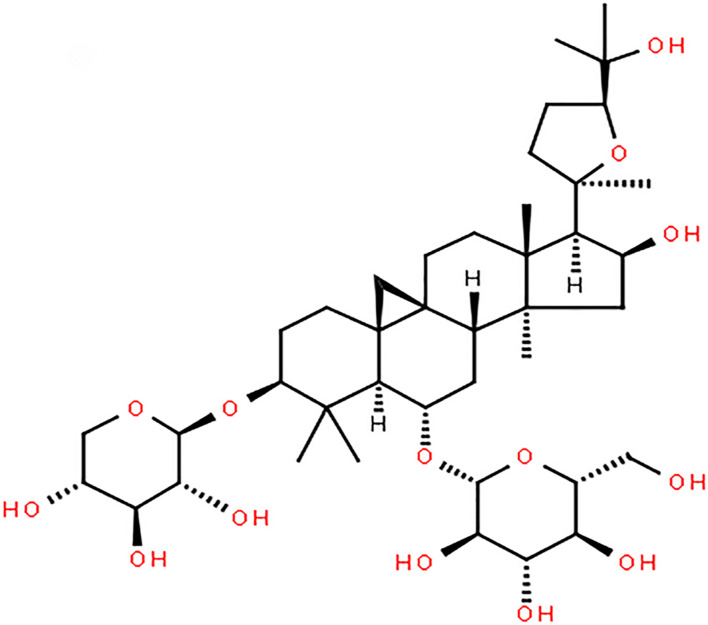
Chemical structure of Astragaloside IV.

Astragaloside IV, a monomer extracted from Radix Astragali (Figure 1; Li et al., 2019), protects brain tissue by inhibiting the expression of peripheral benzodiazepine receptors in the ischemic penumbra and reducing apoptosis. It can also regulate M1/M2 microglia/macrophage polarization and improve the inflammatory response in the ischemic penumbra area, thus protecting brain tissue. Astragaloside IV can ameliorate memory impairment and neuroinflammation in mice with bilateral common carotid artery occlusion by decreasing the expression of Toll-like receptor(TLR)4 and its downstream receptor proteins, including MyD88, TRIF, and TRAF6, and inhibiting the phosphorylation of NF-κB. In addition, astragaloside IV exerts a protective effect on neural stem cells by inhibiting the JNK/C-Jun pathway through miR-138. Other researchers have shown that astragaloside IV can protect the brain against ischemia-reperfusion injury by reducing the permeability of the blood-brain barrier (BBB) under pathological conditions by upregulating Bal-2 expression and downregulating Bax, caspase-3, BIP, CHOP, P-PERK, and P-eif2α expression, thereby reducing endothelial cell apoptosis and inhibiting endoplasmic reticulum stress. Recently, some scholars have confirmed the ability of astragaloside IV to cross the BBB through computational studies, and model analysis has verified the ability of astragaloside IV to cross the BBB (Stȩpnik and Kukula-Koch, 2020). This lays the foundation for the clinical application of astragaloside IV in the future.

## Mechanism Underlying the Amelioration of Ischemia-Reperfusion Injury by Astragaloside IV

There are many mechanisms of ischemia-reperfusion injury: ischemia-induced neuronal apoptosis, oxidative stress (Chan, 1996), BBB damage (del Zoppo and Mabuchi, 2003), leukocyte adhesion to vessel walls (del Zoppo et al., 1991), parenchymal infiltration (Zhang et al., 1994a,b), hemorrhagic transformation, and inflammatory responses triggered by ischemia and exacerbated by reperfusion (Jean et al., 1998; Lindsberg and Grau, 2003; Liu et al., 2011). Several studies have shown that astragaloside IV can alleviate brain injury caused by ischemia-reperfusion through multiple pathways (Table 1).

**TABLE 1:** Protective effect and mechanism of astragaloside IV against cerebral ischemia-reperfusion injury.

No.	Study object/model	Test indicator	Mechanism	Effect	References
1	OGD	(+) Number of surviving cells HK-II expression p-Akt expression (-) Glutamate concentration TUNEL staining DAPI staining PAR expression	(+) Akt phosphorylation (+) Akt binding to HK-II	(+) Hexokinase activity Mitochondrial HK-II (-) Release of proapoptotic proteins and apoptosis-inducing factor (AIF)	Li et al., 2019
2	MCAO SD rats OGD PC12 cells	(+) Cell viability (-) Apoptosis rate of PC12 cells Caspase-3 expression Calcium concentration CaSR expression	(-) CaSR expression	(-)reduce calcium reflux	Du et al., 2021
3	Hypoxia-treated neural stem cells in SD rats	(+) Cell viability Bcl-2 expression miR-138 expression (-) Apoptosis rate Caspase-3 expression Caspase-9 expression Bax expression p-JNK expression p-c-Jun expression p-p38MAPK expression	(+) miR-138 expression (-) JNK/p38 MAPK pathway	(+) Cell survival Anti-apoptotic factor expression (-) Apoptosis rate Pro-apoptotic factor expression	Zheng and Zhao, 2018
4	C57BL/6 mice	(+) Cell survival rate Nrf2 mRNA and protein expression HO-1 mRNA and protein expression	(+) Nrf2/HO-1 pathway	(+) Expression of Nrf2 in nuclei Nrf2 nuclear translocation rate HO-1 expression (-) Expression of Nrf2 in cytoplasm	Huang et al., 2014
5	OGD	(+) Cell viability ATP levels JC-1 expression p-PKA expression p-CREB expression (-) LDH levels ROS levels Caspase-3 expression	(+) PKA/CREB pathway	(+) Nerve cell viability (-) Release of LDH Fragmentation of neuronal fibers and cell bodies Expression of caspase-3	Xue et al., 2019
6	MCAO Bend.3 cells C57BL/6 mice	(+) ZO-1 expression Nrf2 expression HO-1 expression NQO1 expression TEER Occludin expression CLDN5 expression (-) BBB permeability ROS levels VCAM-1 expression IL-1β expression TNF-α expression	(+) Nrf2/HO-1 pathway	(+) Expression of tight junction protein (-) VCAM-1 expression Adhesion of monocytes to vascular endothelial cells	Qu et al., 2009; Li et al., 2018
7	MCAO	(+) Neurological score (-) Infarct size MPO levels TNF-α expression IL-1β expression Number of CD11b/CD18-positive neutrophils ICAM-1 expression NF-κB expression	/	(-) TNF-α and IL-1b production Levels of NF-κB Proportion of CD11b/CD18-positive neutrophils Expression of intercellular adhesion molecule-1 (ICAM-1)	Li et al., 2012
8	BV-2 microglial cells	(+) Cell viability IL-10 expression Arg-1 expression (-) NO expression IL-1β expression IL-6 expression TNF-α expression IL-4 expression TLR4 expression MyD88 expression NF-êB expression iNOS expression	(-) TLR4/MyD88/NF-B pathway	(+) Expression of anti-inflammatory factor (IL-10) (-) LPS-induced M2 to M1 transition in microglia NO production Expression of proinflammatory factors (IL-6, TNF-α)	Yu et al., 2019
9	MCAO SD rats	(+) PPARγ mRNA expression PPARγ protein expression Number of CD206+/Iba1+(M2) cells Number of BrdU+/NeuN+ cells Number of BrdU+/GFAP+ cells Number of BrdU+/vWF+ cells (-) Number of CD16/32+/Iba1+ (M1) cells	(+) PPARγ pathway	(+)M1 microglia/macrophages convert to M2 microglia/macrophages	Li et al., 2021

### Ischemia-Induced Neuronal Apoptosis

**FIGURE 2:**
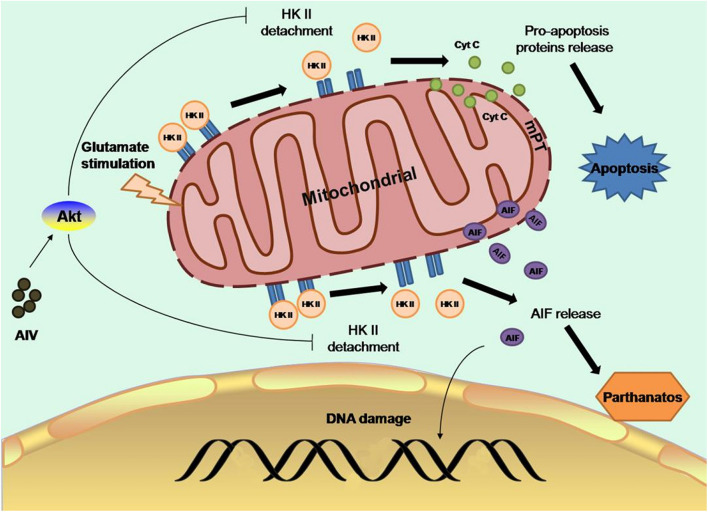
Astragaloside IV can preserve mitochondrial HK-II and subsequently protect neurons from apoptosis and cell death by promoting the binding of Akt to HK-II, which activates Akt and protects mitochondrial HK-II, improving glycolysis, and protecting hexokinase (Springer).

Glutamate-releasing enzyme-mediated excitotoxicity is the main cause of ischemic brain injury (Tymianski, 2011). Elevation of glutamate concentrations in the ischemic area of the brain activates neuronal N-methyl-D-aspartate receptor (NMDAR), mediates extracellular calcium influx, and increases intracellular calcium concentrations, and intracellular calcium triggers the apoptosis cascade, leading to cell dysfunction (Lai et al., 2014). Glutamate stimulation induces dissociation of mitochondrial hexokinase II (HK-II) from mitochondria, resulting in impaired mitochondrial function, as evidenced by opening of the mitochondrial permeability transition pore (mPTP) (Nederlof et al., 2014), collapse of the mitochondrial membrane potential, and decreased neuronal mitochondrial oxygen consumption, accompanied by apoptosis, oxidative DNA damage, PAR formation (Alano et al., 2004), and nuclear translocation of apoptosis-inducing factor (AIF), which is indicative of dependent cell death (Yu et al., 2006). Moreover, calcium overload is an important link between apoptosis and neuronal necrosis. Extracellular calcium can be absorbed and transported to mitochondria during ischemia-reperfusion injury, increasing mitochondrial permeability and promoting oxidative reactions and apoptosis (Yin et al., 2020). CaSR, a G-protein-coupled receptor, plays an important role in maintaining calcium homeostasis and regulating calcium influx (Lu et al., 2010; Figure 2).

Astragaloside IV can preserve mitochondrial HK-II, reduce the release of proapoptotic proteins and AIF, and subsequently protect neurons from apoptosis and cell death by promoting the binding of Akt to HK-II, thus activating Akt to protect mitochondrial HK-II, improving the activity of glycolysis, and protecting hexokinase (Li et al., 2019). It has been found that during cerebral ischemia-reperfusion, the protein expression of CaSR and calcium influx increase. Astragaloside IV can inhibit the protein expression of CaSR after ischemia-reperfusion injury to reduce calcium reflux (Du et al., 2021). Radix Astragali exerts a protective effect not only on neurons but also against ischemic apoptosis of neural stem cells. Previous studies have found that ischemia and hypoxia promote neural stem cell proliferation through a feedback mechanism. Stem cell viability increases after 2 h of ischemia and hypoxia. However, with the prolongation of ischemia and hypoxia, stem cell viability decreases in a time-dependent manner (Li et al., 2007). It has been found that the expression of miR-138 is increased in neural stem cells exposed to hypoxia. miR-138, which plays a critical role in promoting the growth and survival of self-renewing tumor-initiating cells, was previously identified as a molecular marker of glioma stem cells (GSCs) (Chan et al., 2012). It has also been reported that the JNK/C-Jun pathway mediates the effect of miR-138 on hypoxia-induced myocardial apoptosis (He et al., 2013). In a recent study, a 50% decline in stem cell viability was observed after an 8-h ischemic preconditioning regimen. Studies have shown that pretreatment of rat neural stem cells with 2.5 or 5 mg/ml Astragalus polysaccharides for 2 h before treatment can increase cell survival, decrease the apoptosis rate, decrease the expression of proapoptotic factors, and increase the expression of antiapoptotic factors. In this study, the mechanism under the effect of Astragalus polysaccharides was also discussed. miR-138 expression was significantly elevated in the Astragalus polysaccharide-treated group compared with the control group, and the expression of phosphorylated JNK and c-Jun was decreased. In contrast, the protective effect of Astragalus polysaccharides on ischemic neural stem cells was abolished after miR-138 inhibitor treatment, suggesting that the protective effect of Astragalus polysaccharides on ischemic neural stem cells is achieved through upregulation of miR-138 expression in NSCs exposed to hypoxia and inhibition of the JNK/C-Jun pathway (Zheng and Zhao, 2018). This finding provides an experimental basis for the treatment of perinatal hypoxic-ischemic encephalopathy (HIE) with Astragalus polysaccharides.

### Oxidative Stress

After cerebral ischemia-reperfusion, the large number of free radicals produced by the brain have a severely damaging effect on nerve cells, and inhibition of the oxidative stress response is an important means for preventing ischemic injury. AREs are *cis*-regulatory elements in the promoter regions of many important antioxidant genes. Nrf2, as a transcription factor, regulates the basic and induced expression of a large number of antioxidant genes by binding to AREs and is one of the key regulators of endogenous antioxidant defense (Kensler et al., 2007). Studies have shown that activation of the Nrf2/ARE pathway increases the nuclear localization of Nrf2; induces the expression of Nrf2/ARE-dependent genes such as HO-1, NQO-1, and SRXN-1; and attenuates cerebral ischemic injury (Zhang et al., 2017).

A comparison of the reactive oxygen species (ROS) levels in an OGD model before and after the administration of astragaloside IV inhibitors explicitly showed that astragaloside IV can inhibit the accumulation of ROS and that this effect is particularly evident at an astragaloside IV concentration of 50 μM. Real-time PCR showed that when the concentration of astragaloside IV is increased, the expression of the Nrf2/ARE-dependent genes HO-1, NQO-1, and SRXN-1 increases in a dose-dependent manner in an OGD model. After lentiviral shRNA-mediated inhibition of Nrf2 in cortical neurons, the scavenging effect of astragaloside IV on ROS is significantly inhibited, showing that the Nrf2/ARE pathway is required for the antioxidative and neuroprotective effects of astragaloside IV against OGD (Gu et al., 2015). Furthermore, astragaloside IV combined with ginsenoside Rg1 or ginsenoside Rb1 and notoginseng R1, which are ineffective when used alone, can activate the Nrf2/HO-1 signaling pathway to a greater extent after cerebral ischemia-reperfusion to downregulate Nrf2 expression in the cytoplasm, upregulate Nrf2 expression in the nucleus, increase the nuclear translocation rate, and increase HO-1 mRNA and protein expression; thus, the antagonistic effect of astragaloside IV against ischemia-reperfusion and oxidative stress injury is enhanced when the drug is combined with these agents (Huang et al., 2014). This provides meaningful guidance for combining drugs in the clinic to reduce their adverse effects and increase their safety. Can astragaloside alleviate neuronal oxidative stress after ischemia-reperfusion through other mechanisms? A previous study revealed that astragaloside IV acts as a key regulator of NO and angiogenesis through the JAK2/STAT3 and ERK1/2 pathways (Wang et al., 2013). In a recent study, astragaloside IV was shown to activate the JAK2/STAT3 signaling pathways, while the JAK2 inhibitor AG490 was found to reverse JAK2/STAT3 activation and the neuroprotective effects of astragaloside IV during OGD/R (Xu et al., 2020).

Abnormal energy metabolism after cerebral ischemia causes mitochondrial damage, resulting in decreased respiration, excessive ROS production, adenosine triphosphate (ATP) depletion (Fiskum et al., 1999; Perez-Pinzon, 2004), and inhibition of the PKA-CREB signaling pathway. The PKA-CREB signal transduction pathway can promote the survival, regeneration and differentiation of neural cells, and its downstream protein UCP-2 can reduce the mitochondrial membrane potential, resulting in mild mitochondrial decoupling and thus reducing ROS production. However, the exact mechanism by which UCP-2 regulates ROS production remains unclear (Ježek et al., 2018). Recently, the “decoupled survival” hypothesis, which is associated with UCP-2, was further confirmed in models of traumatic brain injury and ischemic stroke (Normoyle et al., 2015).

**FIGURE 3:**
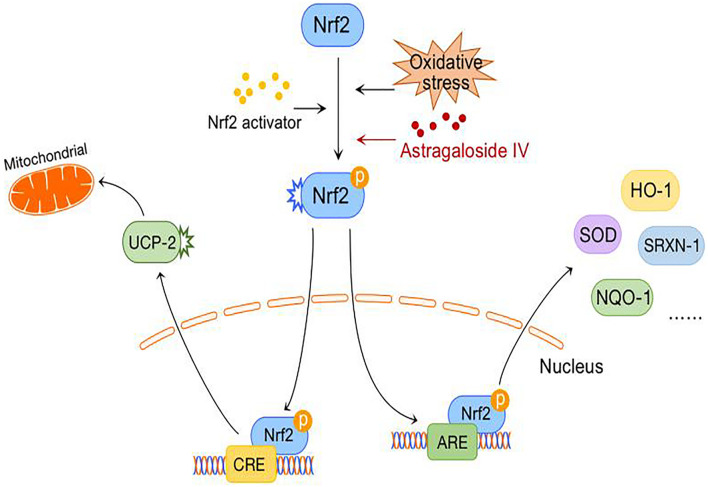
Antioxidative stress mechanism of astragaloside IV.

A previous study found that astragaloside IV might activate the PKA/CREB pathway, increase the mitochondrial membrane potential, and reduce the release of ROS in an OGD model. In contrast, it increases the release of ATP and improves mitochondrial function. Furthermore, astragaloside IV significantly reverses the release of LDH during OGD and the fragmentation of neuronal fibers and cell bodies, reduces the expression of caspase-3, and improves neuronal viability (Xue et al., 2019; Figure 3).

Astragaloside IV increases SOD activity and SOD mRNA expression in astrocytes. Supplementation with astragaloside IV after OGD/R exposure promotes the expression of oxidation and apoptosis markers, and further research has demonstrated that astragaloside IV inhibits the CXCR4 receptor and decreases the activation of the p-JNK/JNK pathway, thus suppressing the expression of Bax/Bcl-2 and ultimately promoting Nrf2/Keap1 signaling (Yang et al., 2021).

### Damage to the Blood-Brain Barrier

The BBB consists of a continuous layer of brain capillary endothelial cells, pericytes, basement membranes, and astrocytes. Tight junctions between endothelial cells form metabolic and physical barriers, which limit the movement of macromolecules between the blood and the brain to maintain homeostasis in the brain (Kago et al., 2006). Following ischemic stroke, tight junction complexes in vascular endothelial cells are altered, causing increased paracellular solute leakage. Regulation of transporters and changes in intracellular transport mechanisms lead to disturbances in the transcellular transport of certain substances (Abdullahi et al., 2018). Therefore, protecting the BBB facilitates neuronal recovery after ischemia-reperfusion injury. Hemorrhagic stroke, such as aneurysmal hemorrhage, can lead to increased intracranial pressure, reduced cerebral blood flow, total cerebral ischemia, cerebral edema, blood component spillage, and decomposition product accumulation; in addition, it can damage the BBB, expose neural tissues to neurotoxic blood and immune cells and result in the development of delayed vasospasm, which leads to poor prognosis of stroke patients. This pathophysiological process may be mediated by TLR4, netrin-1, lipocalin-2, tropomyosin-associated kinase receptor B and the tyrosine kinase ErbB4 (Li et al., 2020).

Lanthanum staining was performed in an ischemia model and a sham operation model to compare the localization of lanthanum. Lanthanum was found in the sham operation group, while it was found in the perivascular tissues in the ischemia group. These results confirm that ischemia leads to dysfunction of the BBB. In the astragaloside IV-treated group, lanthanum was mainly confined to the cerebral capillaries, suggesting that astragaloside IV may maintain the integrity of the BBB in rats subjected to ischemia-reperfusion. A decrease in Evans blue leakage and an increase in the expression of the tight junction protein ZO-1 have also been reported, strongly supporting this idea (Qu et al., 2009). Other studies have found that astragaloside IV has an effect on BBB endothelial cells and inhibits the deterioration of inflammation to reduce the adhesion of JAWS II cells to bEnd.3 cells, decrease the expression of cellular VCAM-1, and increase the expression of cellular tight junction proteins (e.g., zo-1, occludin, and CLDN5). However, Nrf2 siRNA abolishes these effects of astragaloside IV and its protective effect on tight junctions, indicating that the protective effect of astragaloside IV against LPS-induced BBB endothelial cell injury is dependent on the Nrf2 signaling pathway (Li et al., 2018).

Moreover, experiments have demonstrated that astragaloside IV reduces the expression of TLR4 in rats with experimental arachnoid hemorrhage, thus reducing the activation of Nrf2 and decreasing the occurrence of delayed cerebral spasm (Ma et al., 2018). This finding suggests that astragaloside-IV also has a protective effect against delayed cerebral spasm after hemorrhagic stroke.

### Leukocyte Adhesion to the Vascular Wall and Cerebral Parenchymal Infiltration

Inflammatory injury plays an important role in cerebral ischemia-reperfusion injury, and cerebral blood flow is interrupted after arterial occlusion. The acute inflammatory response is caused by neutrophil adhesion to ischemic endothelial cells (Chou et al., 2004; Yilmaz and Granger, 2010). Integrins and the immunoglobulin superfamily play a major role in this process (Huang et al., 2006). CD11b/CD18 is one of the major integrins in neutrophils and can recognize and bind to immunoglobulin superfamily members on the surface of endothelial cells (Springer, 1990; de Fougerolles et al., 1991; Shen et al., 1998).

Astragaloside IV has been found to play a protective role by significantly reducing the production of TNF-a and IL-1b, decreasing the level of NF-κB, significantly reducing the proportion of CD11b/CD18-positive neutrophils, and downregulating the expression of intercellular adhesion molecule-1 (ICAM-1). However, interestingly, this protective effect is not significantly correlated with the dose of astragaloside IV (Li et al., 2012).

### Inflammatory Reaction in the Ischemic Penumbra

M1 microglia/macrophages can be activated by factors such as LPS, IFN-γ, TNF-α, hypoxia, and amyloid β, which increase the synthesis of proinflammatory factors, chemokines, and oxidative metabolites and worsen the inflammatory response, thereby aggravating neuronal injury and death. In contrast, M2 microglia/macrophages inhibit the inflammatory response by secreting cytokines and neurotrophic factors and promote neuronal repair and regeneration (Kanazawa et al., 2017). Activated microglia/macrophages can be detected in the border zone of ischemic lesions 30 min after permanent middle cerebral artery occlusion (MCAO) (Qin et al., 2019), and it has been demonstrated that the expression of the TLR family members TLR2, TLR4, and TLR9 increases after ischemic brain injury. Elevated TLR4 expression leads to activation of the NF-κB pathway and activates microglia/macrophages, allowing them to undergo the transition from the M2 phenotype to the M1 phenotype (Zhao et al., 2017). In addition, peroxisome proliferator–activated receptor γ (PPARγ), which is widely expressed in macrophages and microglia, is a member of the nuclear receptor superfamily and a ligand-activated transcription factor (Straus and Glass, 2007). PPARγ agonists have been reported to increase M2 microglial/macrophage polarization and promote neurogenesis and angiogenesis after cerebral ischemia-reperfusion (Kinouchi et al., 2018). Therefore, the PPARγ receptor is considered an effective therapeutic target for a variety of central nervous system diseases, including ischemic stroke (Cai et al., 2018).

**FIGURE 4:**
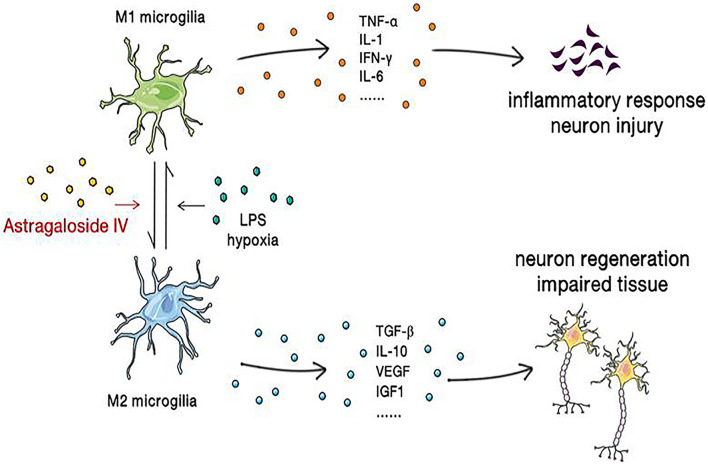
Astragaloside IV reverses microglial polarization, reduces the inflammatory response, ameliorates neuronal injury, and promotes the regeneration of neurons and the repair of brain tissue.

It was found that 5 μmol/l astragaloside IV can inhibit the LPS-induced transition from the M2 phenotype to the M1 phenotype, NO production, and the expression of proinflammatory factors (IL-6, TNF-α) in microglia/macrophages but increase the expression of anti-inflammatory factors (IL-10). Immunofluorescence staining revealed that the expression of TLR4, MyD88, and NF-κB is elevated under stimulation with LPS and that this change can be inhibited by astragaloside IV, suggesting that astragaloside IV exerts its effect through the TLR4/MyD88/NF-κB signaling pathway (Yu et al., 2019). It was recently reported that astragaloside IV is a natural PPARγ agonist (Wang et al., 2017). It was found that astragaloside IV increases the M2 polarization of microglia/macrophages and the expression of PPARγ mRNA and protein. Immunofluorescence staining showed that after administration of the PPARγ antagonist T0070907, the number of CD16/32+/Iba1+ (M1) cells increases, the number of CD206+/Iba1+ (M2) cells decreases, the numbers of BrdU+/NeuN+, BrdU+/GFAP+, and BrdU+/vWF+ cells dramatically decreases and astragaloside IV-mediated neurogenesis and angiogenesis is blocked. The results indicate that astragaloside IV promotes M2 microglial/macrophage polarization through the PPARγ pathway (Li et al., 2021) (Figure 4).

## Protective Effect and Mechanism of Astragaloside IV Against Cerebral Ischemia-Reperfusion Complications

Astragaloside IV also exerts protective effects against cerebral ischemia-reperfusion complications (Table 2).

**TABLE 2:** Protective effect and mechanism of astragaloside IV against cerebral ischemia-reperfusion complications.

No.	Study object/model	Test indicator	Mechanism	Effect
1	MCAO	(+) Neurological score (-) Brain water content BBB permeability MMP-9 expression AQP4 expression	(-) MMP-9 and AQP4 expression	(+) BBB function (-) Water enters astrocytes from microvessels Cerebral edema
2	BCCAO	(+) SOD levels (-) IL-1β expression TNF-α expression MDA levels ROS levels TLR4 expression TRIF expression TRAF6 expression p-P6 expression NLRP3 expression Caspase-1 expression Iba1 expression	(-) TLR4/MyD88, TRIF, and TRAF6/NF-κB pathways	(+) Memory function (-) Excessive activation of astrocytes and microglia Inflammatory reaction

### Cerebral Edema

Cerebral edema after intravenous thrombolysis is a rare but potentially fatal complication (Cruz-Flores et al., 2001) that occurs mainly due to cytotoxic edema resulting from ischemia and vasogenic edema aggravated by reperfusion. The aforementioned disturbance of the BBB caused by ischemia-reperfusion can lead to vasogenic brain edema and play an important role in the course of brain edema. Matrix metalloproteinases (MMPs) are proteolytic enzymes, and MMP-9 expression after cerebral ischemia leads to degradation of several important structural proteins, thus impairing the microvascular wall, increasing microvascular permeability, and disrupting the BBB (Dong et al., 2009; Liu et al., 2009). AQP4 is a member of the transmembrane aquaporin family and the water/glycerol transporter family (Agre, 2006), and AQP4 gene deficiency ameliorates brain edema caused by cerebral ischemia (Manley et al., 2000).

Studies have found that astragaloside IV can reduce the expression of MMP-9 and AQP4, ameliorate BBB dysfunction, and reduce cerebral edema complications. An experiment also revealed that there is no significant correlation between the protective effect and dose of astragaloside IV (Li et al., 2013).

### Memory Impairment

There is a direct relationship between hypoperfusion in the hippocampal CA1 region and memory impairment after cerebral ischemia (Zhang et al., 2010). BBB breakdown (Winkler et al., 2014) and neurovascular dysfunction (Winkler et al., 2015) are also significant pathological features of Alzheimer’s disease. In addition, neuroinflammation also plays an important role in impairing cognitive performance during the progression of neurodegenerative diseases (Harrison et al., 2014).

Astragaloside IV significantly decreases TLR4 expression and the synthesis of downstream adaptor proteins, including MyD88, TRIF, and TRAF6, and then suppresses NF-κB phosphorylation while inhibiting the excessive activation of astrocytes and microglia, alleviating the inflammatory response, and significantly improving memory impairment in mice with bilateral common carotid artery occlusion (BCCAO) (Li et al., 2017). Moreover, as a natural PPARγ agonist, astragaloside IV reduces the formation of neuritic plaques and Aβ plaques by inhibiting the expression of BACE1 to improve cognitive performance in Alzheimer’s disease patients (Wang et al., 2017).

## Limitations and Prospects

The mechanism underlying the cerebroprotective effect of astragaloside IV against ischemia-reperfusion is clear. Astragaloside significantly ameliorates injury caused by ischemia-reperfusion at multiple levels. Astragaloside IV acts on multiple signaling pathways to relieve neuronal apoptosis, oxidative stress, BBB injury, leukocyte adhesion to the vascular wall and parenchymal infiltration caused by ischemia-reperfusion and the inflammatory response triggered by ischemia and aggravated reperfusion to improve brain injury and complications after ischemia-reperfusion and improve prognosis.

However, most of the data have been obtained in cells, rats and mice, as there have been few clinical trials on the effect of astragaloside IV in stroke patients. We searched for articles on clinical trials of astragaloside IV and found that astragaloside IV has ameliorative effects on skeletal muscle injury (Jiang et al., 2020), precancerous lesions of gastric carcinoma (Zhang et al., 2018), liver fibrosis (Wang et al., 2021), induction of natriuresis (Ai et al., 2008), and heart failure (Luo et al., 1995). However, there are very few clinical publication on the protective effect of astragaloside IV on the brain. In addition, astragaloside IV was shown to have a dose-independent effect on some aspects of injury, such as leukocyte adhesion to the vascular wall and parenchymal infiltration.

Astragaloside IV has a certain toxic effect at specific doses, as 10 μmol/l astragaloside IV significantly decreases cell viability (Yu et al., 2019), but the appropriate dose of astragaloside IV can achieve a certain synergistic effect when administered in combination with other drugs such as ligustrazine (Cai et al., 2014) and notoginseng (Huang et al., 2017), providing guidance for clinical practice. Clinically, the use of rtPA for the treatment of acute ischemic stroke is limited not only by the small therapeutic window but also by the occurrence hemorrhagic transformation during ischemia-reperfusion injury. The incidence of spontaneous hemorrhagic transformation after acute ischemic stroke ranges from 13 to 43%, although autopsy results have suggested that the incidence is as high as 38–71%, and the use of rtPA increases this risk by 10-fold (Zhu et al., 2015). However, there is little evidence showing whether astragaloside IV exerts a cerebroprotective effect against intracerebral hemorrhage injury. We need to more comprehensively explore the cerebroprotective effect of astragaloside IV against ischemia-reperfusion through a more in-depth study of astragaloside IV.

## Author Contributions

WG and HT were involved in the study design. WG, HT, and WH provided and prepared the samples. XK and SS wrote the manuscript. All authors contributed to the article and approved the submitted version.

## Conflict of Interest

The authors declare that the research was conducted in the absence of any commercial or financial relationships that could be construed as a potential conflict of interest.

## Publisher’s Note

All claims expressed in this article are solely those of the authors and do not necessarily represent those of their affiliated organizations, or those of the publisher, the editors and the reviewers. Any product that may be evaluated in this article, or claim that may be made by its manufacturer, is not guaranteed or endorsed by the publisher.
